# Digital Health Interventions for Military Members, Veterans, and Public Safety Personnel: Scoping Review

**DOI:** 10.2196/65149

**Published:** 2025-10-28

**Authors:** Rashell R Allen, Myrah A Malik, Carley Aquin, Lucijana Herceg, Suzette Brémault-Phillips, Phillip R Sevigny

**Affiliations:** 1 School and Clinical Child Psychology, Faculty of Education, University of Alberta, Edmonton, AB, Canada; 2 Heroes in Mind, Advocacy and Research Consortium, Faculty of Rehabilitation Medicine, University of Alberta, 8205 114 St NW, Edmonton, AB, T6G 2G4, Canada, 1 780-492-2903; 3 Department of Occupational Therapy, Faculty of Rehabilitation Medicine, University of Alberta, Edmonton, AB, Canada; 4 Counselling Psychology, Faculty of Education, University of Alberta, Edmonton, AB, Canada

**Keywords:** public safety personnel, veterans, military members, mobile apps, web-based programs, resilience, well-being, community, psychological, PTSD, scoping review, PRISMA

## Abstract

**Background:**

Accessible mental health support is essential for military members (MMs), veterans, and public safety personnel (PSP) who are at an increased risk of mental health challenges. Unique barriers to care, however, often leave these populations going untreated. Mental health treatment delivered via apps or websites (ie, digital mental health interventions [DMHIs]) offers an accessible alternative to in-person therapy.

**Objective:**

We aimed to synthesize the current literature on apps and web-based programs focused on enhancing MMs’, PSPs’, and veterans’ resilience or well-being. A multidimensional well-being model, I-COPPE (interpersonal, community, occupational, physical, psychological, economic, and overall well-being), was used as a framework guiding the scoping review.

**Methods:**

A search of 6 databases was conducted using key terms related to (1) population, (2) resilience and well-being constructs, and (3) web- or mobile-based programs. At all levels of screening, at least 2 researchers (RRA, MAM, and CA) reviewed each paper independently. Data were extracted and recorded to include relevant study characteristics including program name and description, target population, number of participants, therapeutic approach, results, limitations, and I-COPPE dimension supported. A narrative synthesis was performed to summarize the eligible studies.

**Results:**

In total, 44 papers were included in the study and 39 unique resilience or well-being apps or web-based programs identified for MMs, PSP, or veterans. The programs largely focused on veteran populations (28/44, 64%). In total, 51% (20/39) of programs relied on cognitive behavioral approaches and most aimed to support posttraumatic stress disorder–related symptoms. In consideration of the I-COPPE model, a majority supported psychological well-being, followed by interpersonal and physical well-being. Most apps were believed to support more than 1 domain of well-being. The main methodologies used in the literature to evaluate digital mental health interventions include randomized controlled trials, secondary analyses, and pilot randomized controlled trials with evaluations of feasibility, acceptability, satisfaction, or qualitative feedback. Generalizability of findings was commonly limited by attrition rates and small sample sizes.

**Conclusions:**

DMHIs for MMs, PSP, and veterans appear promising due to their accessibility and scalability. More research is needed, however, to determine whether DMHIs are an effective alternative to in-person mental health care. The current review contributes to the literature by compiling evidence of DMHIs and the domains of well-being supported by, and the therapeutic orientation of, these programs. Our review revealed that more research is needed to determine the effectiveness and efficacy of DMHIs offered to these populations.

## Introduction

### Background

Military members (MMs) and public safety personnel (PSP; ie, police officers, paramedics, firefighters, correctional officers, peacekeepers, and emergency medical technicians [1]) stand ready and resilient to protect civilians at a moment’s notice. These individuals, including veterans who have completed their military service, have been grouped in this paper due to similarities across their public service work. These individuals, as a result of their work, typically turn toward danger when most turn away. Although there may be potential worldview and cultural and day-to-day task differences across these populations, they have been grouped due to their increased risk of exposure to potentially psychologically traumatic events (PPTEs) as a result of their work [2-4]. Such events may include direct or indirect exposure to death (accidental or violent), transportation accidents, assaults, natural disasters, and military conflict [2,5-7]. PPTE exposure is associated with an increased risk for mental health concerns, including but not limited to posttraumatic stress disorder (PTSD), generalized anxiety disorder, and depression [2,8,9]. The occupational hazards of PPTEs and other occupational stressors such as family separations, relocations, and the long, unpredictable work hours often experienced have implications for well-being and family life [10,11]. Such professional expectations combined with occupational stressors can have negative implications for well-being and thus impact their readiness for occupational duties.

A systematic review (*k*=11) revealed that approximately 29% (55,336/189,021) of MMs who reported a recent mental health concern accessed or sought out services [12], with similar rates observed for veterans [13]. Barriers to service access for PSP, MMs, and veterans include fear of experiencing worsening symptoms, stigma and confidentiality breaches, and avoidance often characteristic of PTSD, as well as practical barriers related to availability of treatment, remote and shift work, transportation, and relocation [13-17]. In addition, the COVID-19 pandemic highlighted the need for greater investment in technology-delivered mental health interventions, referred to as digital mental health interventions (DMHIs) [18-20]. The measures taken for infection control (ie, enforced restrictions, such as social distancing) subsequently reduced access to protective factors (eg, social support networks), contributed to worsened mental health, and interrupted the delivery of in-person treatment [21,22]. Such barriers necessitate specialized mental health services for PSP, MMs, and veterans that are private, accessible, secure, and effective.

Given the accessibility of DMHIs, there are several advantages for their use by MMs, PSP, and veterans. DMHIs offer portable, low-cost, accessible, convenient, personalized, scalable, and self-guided solutions to enhancing well-being [23,24]. Moreover, DMHIs are amenable to inconspicuous use and customization to user preferences and symptomatology [25]. There has been a shift toward DMHIs [26], a promising method for delivering patient-centered health interventions based on people’s present needs [27,28]. Notably, however, in a scoping review of apps for MMs and PSP, only 50% (11/22) were subject to a randomized controlled trial (RCT) [29]. Additional limitations of the apps were noted, such as small sample sizes, limited generalizability of findings, uncontrolled evaluations, and limited follow-up periods [29]. Tam-Seto et al [30], in another scoping review of mental health apps for MMs and veterans, have also reported a lack of research evidence. Based on their review, Tam-Seto et al [30] reported that these populations show a general willingness to use apps, and they suggest that apps may be a supplement to traditional treatment and could be a desirable option for those who fear stigmatization. At this time, although there are some advantages noted for DMHIs, there appears to be insufficient empirical support [31-33]; however, a more recent systematic review of meta-analyses for the general population showed more promising results [34]. For example, Goldberg et al [34] looked at mobile apps, interventions with components of smartphones, and app use with additional equipment and found 14 meta-analyses that met criteria for their review. Of the 14 meta-analyses, 8 showed highly suggestive effect sizes, 4 suggestive, 14 weak, and 8 non-significant [34].

While DMHIs offer many advantages, apps and web-based programs tend to focus solely on enhancing psychological resilience, as opposed to viewing resilience as a multifaceted and dynamic construct [35,36]. Researchers recognize that resilience interventions may lead to positive spillover effects or *collateral change* [37,38]. Such spillover effects, or the ways in which systems influence the resilience of other distinct systems, can be accounted for by a multisystemic resilience perspective [39,40]. Therefore, we define resilience as a system’s process of adaptation (eg, individuals, groups, and organizations) following adversity or risk exposure. Resilience is a dynamic process influenced by socioecological system interactions and available resources, which function as protective factors and lead to improved outcomes when coping with and overcoming adversity [41-43].

Given the importance of well-being in our ability to adapt to stress [44], researchers have started to endorse models wherein improved well-being leads to greater resilience [44,45]. Traditionally, 2 major dimensions of well-being include eudaimonic well-being (behaving virtuously or *functioning well*) and hedonic well-being (*feeling good*) [46]. A multidimensional and multilevel perspective of well-being is the degree to which individual, dyadic, community, and organizations’ needs are satisfied [47,48]. Prilleltensky [49] proposed 7 dimensions of well-being: interpersonal, community, occupational, physical, psychological, economic, and overall well-being (I-COPPE). Given that resilience is multiply determined [40,44], well-being interventions may promote resilience through the enhancement of resources, capacities, and coping skills [37,40]. Consideration of multidimensional well-being and its effects on resilience responds to the call for resilience researchers to expand their focus from remediation of mental distress to positive adaptation and protective processes across multiple levels of influence [50].

A multidimensional focus on well-being is conducive to a *whole-person* approach, which recognizes individuals’ diverse needs that may influence resilience trajectories. As a result, interventions should aim to strengthen protective factors and bolster the development and use of resources to promote resilient responding [45,51-53]. Conversely, the loss of cardinal resources (eg, shelter, safety, and physical well-being) may limit the degree to which individuals benefit from interventions [51,54,55]. While a number of variables are consistently linked to individual resilience trajectories, no particular variable exerts a dominating influence [35], as such interventions should strive to strengthen protective networks [53].

### Purpose

Two relevant scoping reviews have been published recently. First, Voth et al [29] completed a review of resilience-based apps and programs for MMs and PSP, with 32 studies meeting inclusion criteria. Second, Tam-Seto et al [30] completed a scoping review of mental health mobile apps for MMs and veterans, with 35 papers meeting inclusion criteria. Since the publication of scoping reviews by both Voth et al [29] and Tam-Seto et al [30], there has been a greater reliance on DMHIs as a result of the COVID-19 pandemic [56,57], necessitating an updated review. Our scoping review differs from that of Voth et al [29] and contributes to the literature by (1) including both well-being- and resilience-based DMHIs, (2) including 34 new search terms, (3) using a multidimensional well-being framework to describe the DMHIs and potential resilience spillover effects, and (4) including 3 years of novel research published after their search, which also captures new DMHI research during and post–COVID-19 pandemic. Our scoping review also differs from that of Tam-Seto et al [30], who focused exclusively on apps for MMs and veteran populations, by including PSP populations and web-based programs in our search. No study to date has reviewed well-being and resilience DMHIs for MMs, PSP, and veterans. The findings will be synthesized with respect to the I-COPPE model [58], given the proposed upstream effects of well-being interventions on resilience trajectories [37]. We aim to explore the quality of and to highlight the benefits, gaps, and limitations of peer-reviewed literature on independently used DMHIs (ie, without mental health professional, peer, or researcher support or guidance).

## Methods

### Design of the Review

We initially based our search on similar concepts to Voth et al [29]; however, using an iterative process, we added 34 new search terms, including *front-line worker, veteran, well-being, emotion regulation, internet or online intervention or program, mental health app, and mobile app* (see Multimedia Appendix 1 for full list of search terms). The PRISMA-ScR (Preferred Reporting Items for Systematic Reviews and Meta-Analyses extension for Scoping Reviews) was used to inform decision-making and paper identification [59]. No registered review protocol exists for the current project; however, the information provided may be used to replicate the current findings.

### Eligibility Criteria

Literature included in the search encompassed studies aimed at capturing app- and web-based resilience and well-being interventions for MMs, PSP, and veterans (see Textbox 1 for eligibility criteria). Eligible studies evaluated app or web-based resilience and well-being interventions for MMs, PSP, and veterans. The authors defined a web-based program as an *interactive online platform* that does not involve an invitation or log-in code.

Textbox 1. **Eligibility criteria for papers included in the study.**

**Inclusion criteria**

- Self-directed app or web-based intervention meant to improve well-being or resilience.
- The study population included military members (MMs), veterans, public safety personnel (PSP), and students or individuals in training for these professions. Studies focusing on MMs, veterans, and PSP family units were also eligible for inclusion.
- Primary analysis (ie, quantitative analysis and qualitative), including studies evaluating app or program effects, acceptability, usability, feasibility, or change over time.
- English papers published from 2000 to December 13, 2022.
- The study included an app-based or web-based program related to and intended to improve resilience or well-being (ie, substance use cessation, sleep coaching, yoga, social, mental health, physical health, and mindfulness).

**Exclusion criteria**

- The intervention included a guided (synchronous) support component for the intervention (eg, in-person or virtual check-ins [or sessions], therapist interaction, virtual reality, or in-person meetings or discussions).
- App or programs targeting MMs, PSP, and veteran family members, without also focusing on the MMs, PSP, or veteran. App or programs intended only for service provider use with clientele.
- Study is not a primary analysis (eg, single-subject studies, review papers, meta-analyses, research proposals or protocols, and abstract or conference submissions).
- Non-English papers published before 2000 and after February 27, 2024.
- Intervention involved additional technology related to the intervention (eg, heart rate variability monitors and wearable technology).

#### Inclusion Criteria

To capture the increase in apps and websites available and technological innovations during this time, this study included literature published from 2000 to February 27, 2024. In addition, studies meeting inclusion criteria examined DMHIs through primary analyses and focused on PSP, MMs, or veteran populations.

#### Exclusion Criteria

Research on synchronous interventions was excluded, given our aim is to evaluate asynchronous apps and programs. More specifically, we aimed to evaluate DMHIs that were not potentially mediated by therapist, peer, psychologist, or researcher support and guidance. Therefore, any DMHI study that included peer, therapist, psychologist, or researcher support was excluded to allow us to review the literature that solely looked at DMHI usability, acceptability, feasibility, effectiveness, or efficacy that was not potentially mediated by another support person. Studies that included 2 treatment conditions, 1 with additional support and 1 without additional support, were included in this study. DMHIs requiring additional technology (beyond a mobile or computer device) were also excluded for the same reason. Finally, studies on DMHIs aimed at supporting MMs, PSP, or veteran families that did not include a focus on the MMs, PSP, or veterans were excluded.

### Search Strategy and Information Sources

To identify relevant literature, a search (using a Boolean format) was conducted using key terms based on three concepts: (1) population (eg, military, veteran, or PSP), (2) resilience and well-being–related constructs (eg, hardiness or grit), and (3) web- or mobile-based programs (eg, game or apps). The final search was conducted on February 2024 of the following databases: Academic Search Complete, CINAHL, APA PsycINFO, Embase, SocINDEX, and MEDLINE.

### Eligibility Assessment and Study Selection

Three researchers (RRA, MAM, and CA) were involved in the eligibility assessment and study selection, with at least 2 reviewing each article independently. The researchers initially met to discuss and review the eligibility criteria and conducted a prescreening with 12 papers to assess the comprehensiveness and overall agreement of the search criteria. There was disagreement on 1 paper, which was then discussed in-depth, leading to further clarification of eligibility criteria. At all levels of screening (eg, title, abstract, and full-text review), a minimum of 2 researchers reviewed each paper independently. All abstracts with discrepant ratings were included for further review during full-text screening. During the full-text review, conflicts were reviewed by a third reviewer (RRA, MAM, or CA) and discussed in a research team meeting. Following this, final eliminations and decisions were made. Any discrepancies in agreements were recorded for the purpose of calculating interrater reliability.

### Charting the Data

Data were extracted and recorded by a minimum of 2 researchers (RRA, MAM, and CA) for each study to include relevant study characteristics: app or program name and description, target population and number of participants, facets of resilience they purport to support, methods used to support resilience, results, gaps in service or limitations, and I-COPPE dimension supported. Since scoping reviews are meant to be descriptive as opposed to evaluative [60], eligible studies were not assessed in terms of quality. Instead, a narrative synthesis was performed by 3 researchers (RRA, MAM, and CA) to summarize the eligible studies. Specifically, the findings from multiple studies were summarized and explained descriptively. After 2 researchers (RRA, MAM, or CA) charted the data independently, a third reviewer (RRA, MAM, or CA) synthesized the data and identified and rectified any discrepancies between the previous researchers to minimize potential errors. The researchers then met to discuss the resulting data synthesis and core content themes that emerged based on the data extraction. The I-COPPE model [58] provided a theoretical framework for the synthesis, whereas the cascading resilience model [37] represents the underlying theory illuminating how resilience may be supported in these interventions.

## Results

### Study Selection

**Figure 1.**
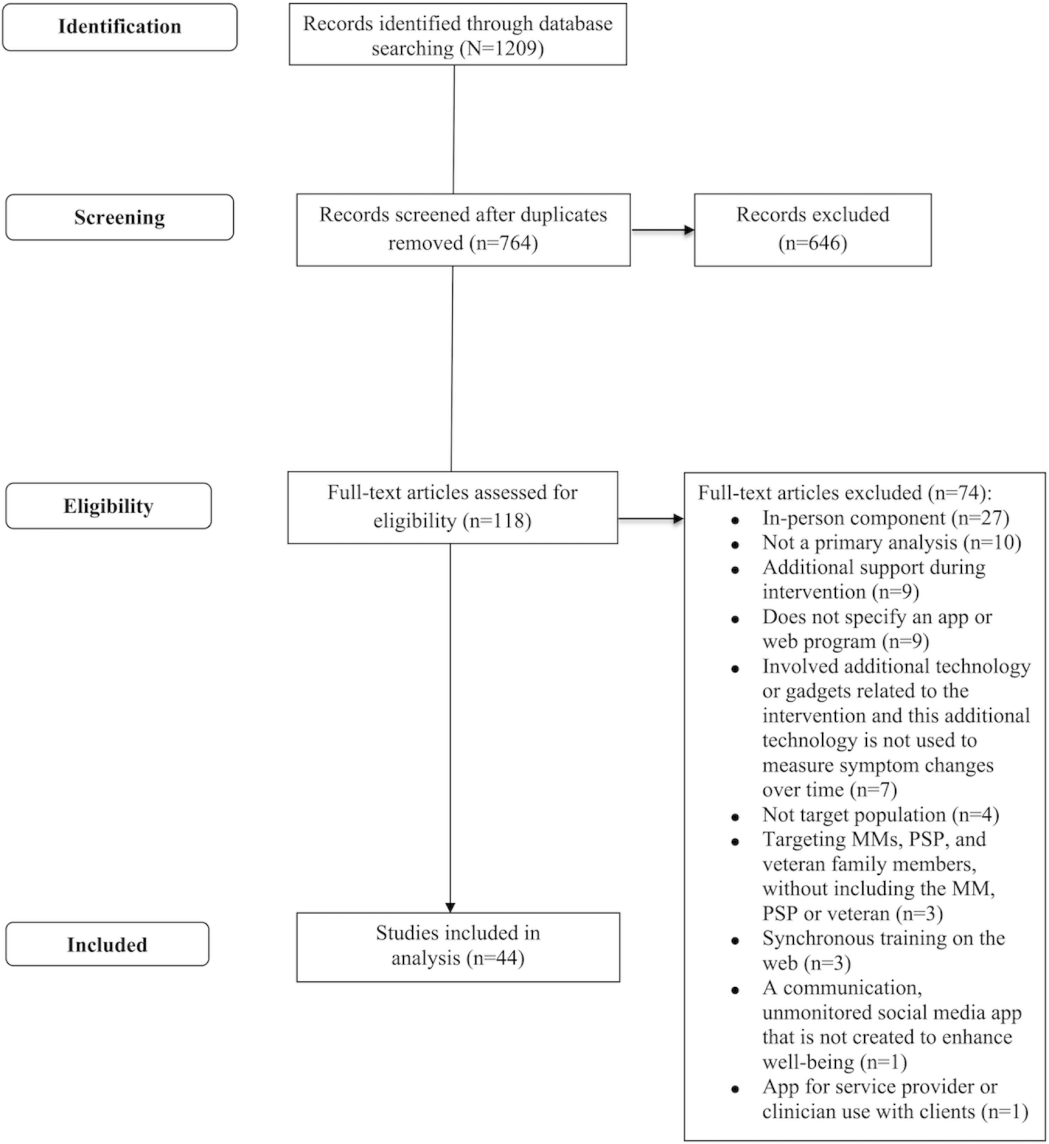
PRISMA (Preferred Reporting Items for Systematic Reviews and Meta-Analyses) flowchart. PSP: public safety personnel.

Details of the screening process can be found in the PRISMA (Preferred Reporting Items for Systematic Reviews and Meta-Analyses) flowchart (Figure 1). A total of 1209 papers were identified, with 434 duplicates, resulting in 775 papers. Three researchers completed initial reviews of titles and abstracts with a range of agreement (Cohen κ) from moderate (κ=0.48) to substantial (κ=0.79). All papers with disagreement were included in the full-text review to allow for a more comprehensive evaluation of the discrepancies. In the end, 118 papers met criteria for full-text review.

Agreement for full-text reviews was fair (κ=0.20) to substantial (κ=0.79). One researcher (MAM) contacted 8 authors because the DMHI name was not specified in reviewed papers. Five studies were excluded due to no response. Three authors responded [61-63], and upon further review, 2 studies met criteria [61,62] whereas 1 [63] did not because the program included various unnamed resources from the Veterans Affairs website. Finally, 1 study [64] was included on the merit that the program would indirectly help veterans through enhancing support strategies provided by their spouse or partner. In the end, 44 papers were included in the study.

### Narrative Synthesis

#### Characteristics of Studies and Populations

Of the 44 papers reviewed, a majority (35/44, 80%) were written by authors residing in the United States and 73% (32/44) were published from 2020 onward, with only 7% (3/44) of papers published prior to 2013 (Table 1).

Most programs focused on veteran populations (28/44, 64%), followed by PSP (8/44, 18%) and MMs (5/44, 11%). Across populations, substance use and PTSD symptomatology were the most common presenting concerns targeted by the programs ( Table 2).

**Table 1.** Summary of study location and publication year (N=44).

Paper information	Values, n
Location of study	1
Canada	1
United States	35
United Kingdom	3
Switzerland	1
Germany and Switzerland^a^	1
Netherlands	1
Australia	2
Publication year	
2022 to February 2024	8
2020‐2022	13
2017‐2019	11
2014‐2016	9
2013 or earlier	3

a The study was conducted in both Germany and Switzerland.

**Table 2.** Summary of key paper information (N=44).

Paper criteria	Values, n
Population and presenting concerns	
Family, friend, or partner of veterans	1
Family member of veteran	1
With PTSD^a^	1
Military members	2
With behavioral health issues	2
Military members and veterans	2
With PTSD symptoms	2
Public safety personnel	8
Firefighters	2
Medical or emergency personnel (ie, police and firefighters)	2
Ambulance workers and firefighters	1
Health care professionals	1
With PTSD symptoms	1
Police officers or students	1
Student paramedics	1
Veterans	28
With PTSD symptoms	5
With PTSD symptoms and substance use	5
With substance use	5
With suicidal ideation	3
With chronic musculoskeletal pain	2
With lower back pain	2
With mental illness (borderline personality disorder, PTSD, bipolar disorder, or depression)	2
With history of chronic mental illness and psychosis symptoms	1
With history of mild traumatic brain injury	1
With insomnia symptoms	1
With substance use and sleep problems	1
Veterans and their nonveteran partners	1
DMHI^b^ as stand-alone or supplementary to therapeutic care	
Supplementary to therapeutic care	5
Not supplementary to therapeutic care	39

a PTSD: posttraumatic stress disorder.

b DMHI: digital mental health intervention.

#### Characteristics of DMHIs

While Voth et al [29] reviewed a total of 32 papers and 22 apps, this study evaluated 44 papers and 39 DMHIs (see Table S1 in Multimedia Appendix 2 for a list of the DMHIs and their characteristics [23,45,61,62,64-103]). Out of the programs evaluated, 16 were assessed as web-based programs, 22 were examined as apps, and 1 was evaluated as both an app and a web-based program. In total, 20 programs relied on cognitive behavioral approaches (ie, cognitive behavioral therapy, exposure therapy, and stress inoculation). Ten of the apps used either psychoeducation or self-management, and 5 programs used motivational interviewing or applied behavior change theory. Five programs emphasized social skills or engagement, several programs focused on regulation strategies (eg, relaxation, stress management, and emotion regulation), and 5 focused on mindfulness as a therapeutic approach. Few programs emphasized social cognitive theory and self-monitoring. The least common therapeutic approaches were SMART goals, sleep restriction therapy, and massage, as well as biopsychosocial, patient-centered, and strengths-focused approaches. Four apps did not specify their therapeutic approach.

Of all 44 papers included in the scoping review, 30% (13/44) were RCTs and 20% (9/44) pilot RCTs (most with evaluations of feasibility, acceptability, satisfaction, or qualitative feedback). Other common research designs included secondary analyses of studies already included (6/44, 14%), quasi-experimental (4/44, 9%), multiphase (eg, app development, feedback, and feasibility; 3/44, 7%), pilot quasi-experimental (2/44, 5%), and feasibility or acceptability (2/44, 5%; Table 3). Other methodological approaches in the DMHI literature for MMs, PSP, and veterans included pilot study for user satisfaction, perceived helpfulness, and usage patterns, mixed methods, and proof of concept.

**Table 3.** Summary of study methodologies (N=44).

Paper criteria	Values, n
Study design	
RCT^a^	13
Pilot study	11
RCT and feasibility, acceptability, satisfaction, or qualitative	6
RCT	3
Quasi-experimental	2
User satisfaction, perceived helpfulness, and usage patterns	1
Secondary analysis of study already included	6
Quasi-experimental design	4
Multiphase (eg, app development, feedback, and feasibility)	3
Feasibility or acceptability trial	2
Mixed methods	1
Proof of concept	1
Qualitative	1
Repeated-measures design	1

a RCT: randomized controlled trial.

### I-COPPE Domains

All papers were examined and synthesized with consideration of the I-COPPE model [58] of well-being. The majority of apps were judged to support more than 1 domain of well-being (see Table S1 in Multimedia Appendix 3 for summary). Programs conceptualized within the psychological well-being domain (97%, 38/39) featured themes of mental health symptom management, treatment modalities such as cognitive behavioral therapy and dialectical behavioral therapy, self-efficacy, and motivation for change. Of the 39 programs included, the most commonly targeted psychological proximal and distal factors were symptoms of PTSD (22/39, 56% of programs); depression (14/39, 36%); resilience (12/39, 31%); self-efficacy, coping, or emotion regulation (10/39, 26%); and quality of life (8/39, 21%). Papers that were grouped within the interpersonal well-being dimension (21/39, 54%) tended to emphasize the development of social support networks for support in mitigating physical and mental health symptoms. A common interpersonal proximal and distal factor targeted and evaluated was communication (4/39, 10%). Apps and programs that fell within the physical well-being domain (21/39, 54%) emphasized reduction of physical symptoms related to sleep difficulties, substance use, PTSD (ie, elevated heart rate), and concussion. Management of psychosis, substance abuse, and pain were categorized within this domain, given their associated physical health implications. Studies categorized under the overall well-being dimension (3/39, 8%) included holistic measures of quality of life and life satisfaction. For community well-being (2/39, 5%), these papers and programs focused on promoting participants’ community engagement and support. Papers supporting occupational well-being (1/39, 3%) concerned occupational support, guided communication, reducing stigma, and greater support services and resources. The economic domain was not captured within the current literature.

### Results of DMHI Programs

Overall, program use typically led to reported improvements in these areas; however, few reported statistically significant changes as compared with treatment-as-usual or control groups (see Table S1 in Multimedia Appendix 4 for summary of study methodologies and results [23,45,61,62,64-103]). The programs that reported positive efficacy results via RCT were (1) Concussion Coach for decreasing postconcussive symptoms [65], (2) Family Foundations for decreasing parent depression [61], (3) Mission Reconnect for improving sleep quality and response to stressful experience [66], (4) Resilience@Work for facilitating coping behaviors (ie, optimism, use of instrumental and emotional support, and active coping) [67], (5) Support Coach for increasing psychological resilience [68], (6) Thinking Forward for decline in perceived alcohol consumption [69], (7) VetChange for improving drinking behaviors and PTSD symptoms [70], and (8) Virtual Hope Box for facilitating coping with unpleasant thoughts or emotions [71].

### Treatment as Stand-Alone or With Support

Some mobile apps and web programs differed in whether they were examined as stand-alone interventions, or whether participants received additional support. Most DMHIs were self-guided. Studies on T2 Mood Tracker [72], Virtual Hope Box [71,73,74], PTSD Coach [75], and Information about Drinking in Ex-serving personnel [23] were supplemented by clinician support, feedback, or clinical monitoring. Other nonclinician supplementary care was provided by peers [76], research staff [77], or mental health specialists [78]. Four papers did not assess intervention effectiveness based on group membership (ie, support vs no support) [23,72,74,78]. For PTSD Coach [75], both groups (ie, those with or without additional support) experienced significant reductions in PTSD symptoms; however, no significant group differences emerged. Those in the clinician-supported group were more likely to attend an additional PTSD session and accept a treatment referral [75]. For Virtual Hope Box, no differences emerged between treatment groups (treatment-as-usual with or without Virtual Hope Box) were reported for enlisting support from friends and family, suicidal ideation, reasons for living, coping self-efficacy, and suicidal ideation [71,73]. For support offered via check-in versus self-managed groups, no statistically significant group differences emerged [77]. Finally, in the feasibility pilot study of Thinking Forward, Possemato et al [76] found no significant difference between peer support and self-managed groups for alcohol use, PTSD symptoms, resiliency, social quality of life, coping, and psychological quality of life.

### Mediators and Moderators

A number of moderators and mediators were considered across the studies to assess their impact on post–program outcomes, engagement, and program effectiveness. Several moderators were statistically significant, including parent gender [61], PTSD symptoms [79], combat exposure [79], self-efficacy [65], and interpersonal problems [80]. More specifically, there was a dosage effect for father-reported parenting undermining, but not for mothers [61]. Brief et al [79] found that there was a sharper decline in drinking behaviors for individuals with higher levels of baseline PTSD symptoms and baseline combat exposure. Belanger et al [65] discovered a greater probability for reduction in PTSD symptoms and psychological distress when self-efficacy was increased. Finally, Polizzi et al [80] found that individuals who reported higher interpersonal problems as a result of their drinking at baseline demonstrated greater PTSD symptoms at baseline and exhibited greater reduction in PTSD symptoms postintervention than those with moderate and low interpersonal problems. In terms of mediators, Williams et al [81] reported significant mediators for 2 apps. For Drinker’s Check-Up, perceived norms of same-age peers for quantity of drinks and number of drinking occasions were mediators negatively and significantly impacting participants' alcohol use behaviors [81]. For Alcohol Savvy, perceived norms (of same-age peers) regarding number of drinking occasions were a mediator negatively and significantly impacting participants' alcohol use behaviors [81].

### Gaps and Limitations

From the 38 studies, there appeared to be recurring limitations for the research reviewed. Two of the most commonly cited limitations were difficulties with attrition (attrition rates ranging from 0% to 82%) and small sample size, limiting the generalizability of findings. In addition, the majority of studies used self-report outcome measures and mentioned concerns with the psychometrics of the measures used. Of the 14 of 42 (32%) papers that mentioned or directly measured resilience, only 5 provided an operational definition of resilience. Features of study design were reported as a limitation by 57% (22/39) of studies with concerns regarding lack of random assignment, blinding, control group, and control for confounding variables. A number of studies (11/39, 29%) also made a call for future research to increase the duration of intervention and follow-up period. Finally, many studies reported concerns regarding app features, app accessibility, and study design.

## Discussion

### Principal Findings

This study reviewed 44 papers and 39 DMHIs. We identified 9 of the same apps compared with the review by Voth et al [29] and 30 new apps and web-based programs compared with their search. Out of the 39 DMHIs, 16 are web-based programs, 22 are apps, and 1 is available as an app and web-based program. Notably, 21 new papers have been published since 2020, highlighting the growth of apps and web-based programs for these populations and justifying the need for this updated review. The majority of the studies took place in the United States and were published between 2020 and 2024. Most studies recruited veteran populations, suggesting a need for apps and programs for MMs and PSP populations.

### I-COPPE and Cascading Resilience

The DMHIs included in this study were organized by the authors based on domains of well-being perceived to be supported (ie, the I-COPPE model). In total, 97% (38/39) of the DMHIs supported psychological well-being. This is not surprising, given that resilience and well-being are often conceptualized as the lack of mental health symptomatology or diagnosis [104]. Although psychological well-being is defined as satisfaction with one’s emotional life [58], this literature tended to focus on decreasing distal outcomes (eg, reducing symptoms) as opposed to promoting proximal factors (eg, self-efficacy or coping to remediate symptoms). Targeting proximal processes may instigate positive cascades across systems [37]. For example, Pavlacic et al [105] found that MMs and veteran populations with higher coping behaviors and self-efficacy were more likely to exhibit a resilience response, emphasizing that targeting proximal factors may be an effective intervention approach.

 It is also notable that there were a high number of apps and web-based programs that supported physical (21/39, 54%) and interpersonal (21/39, 54%) well-being. Both well-being domains are key for functioning well, feeling good, and exhibiting a resilience response [105,106]. Pavlacic et al [105] found that MMs and veterans who reported alcohol problems, poorer physical health, smoking, and presence of a sleep disorder were less likely to exhibit a resilience trajectory and were more likely to exhibit symptomatology. Conversely, veterans with lower self-reported physical health difficulties were more likely to exhibit a resilient response [107]. These results suggest that physical well-being is closely related to resilience and an imperative area to target in a holistic approach to intervention. In terms of interpersonal well-being, MMs and veterans who reported decreased societal exclusion at home and increased social support were more likely to exhibit a resilience response [105]. In a similar study with veterans, Pietrzak and Cook [107] found that individuals with positive reports of social connectedness and social engagement were more likely to respond in a resilient way. Therefore, by targeting physical and interpersonal well-being, there are likely to be positive well-being and resilient responses, as well as potential positive spillover effects across other domains of well-being.

A limitation of the DMHI research, from a whole-person multisystemic resilience framework, is that community, occupational, and economic well-being were each emphasized by 4 or fewer programs. Clearly, greater consideration for community, economic, and organizational well-being is needed [48,58], especially as enhanced well-being across these domains may lead to increased likelihood of a resilience response [58] and positive spillover effects, such as improved resilience across systems [40,108]. Individuals with higher levels of protective psychosocial characteristics related to community well-being and overall well-being (eg, purpose in life), and positive perceptions of the military’s effect on one’s life (ie, occupational well-being) are more likely to exhibit a resilient response when faced with a number of PPTEs [107]. Economic well-being is posited to be closely related to satisfaction with one’s financial situation and is closely related to physical and psychological health [58]. Therefore, by supporting one’s economic well-being, there are likely spillover effects into psychological and physical well-being, which likely also have positive impacts on one’s ability to positively engage in work, with the community, and with important people in their lives.

### Critical Analysis of DMHIs: Strengths and Weaknesses

Most studies (40/44, 91%) specified a therapeutic orientation, with 51% (20/44) purporting a cognitive behavioral framework. Problematically, 9% (4/44) of studies did not provide a therapeutic approach or framework for their intervention. It is important to frame an intervention within a therapeutic framework or approach as this acts as a road map to understand presenting problems and how potential treatments may present solutions to these problems [109]. Not including a therapeutic framework may introduce an array of pitfalls in terms of implementation and evaluating program effectiveness [109]. The most common presenting problems in the populations were PTSD symptoms and substance use behaviors, and the most commonly targeted proximal and distal factors were PTSD, depression, and resilience. Most DMHIs were evaluated in terms of symptom reduction, and few were evaluated in terms of their ability to improve protective factors, indicative of the proclivity toward a diagnostic approach. This emphasis on pathology over resilience-supporting processes is problematic [110]. By evaluating processes, researchers may have an improved understanding of the extent to which DMHIs support well-being and subsequent resilience cascades. Future research would benefit from a process-based orientation and longitudinal methodologies to investigate the process of DMHIs on individual resilience trajectories [110-113]. A longitudinal approach can provide insights into healthy trajectories [112] and illuminate whether and how certain protective factors impact resilience trajectories [104].

An area for growth in the DMHI research is the need for RCTs. These rigorously controlled studies are necessary as they evaluate the impact of interventions with high levels of internal validity and determine the efficacy of the intervention [114,115]. In the current review, only 30% (13/44) of the studies were RCTs and 20% (9/44) were pilot RCTs (most with evaluations of feasibility, acceptability, satisfaction, or qualitative feedback). The pilot RCTs are promising such that they may lead to full efficacy trials, and the initial results illuminate some positive results. More specifically, there were 8 studies that reported positive efficacy results, including Concussion Coach (decreased postconcussive symptoms), Family Foundations (decreased parent depression), Mission Reconnect (improved sleep quality and response to stressful experience), Resilience@Work (improved coping behaviors), Support Coach (increased psychological resilience), Thinking Forward (decreased perceived alcohol consumption), VetChange (improved drinking behaviors and PTSD symptoms), and Virtual Hope Box (improved coping with unpleasant thoughts or emotions). Despite methodological strengths, RCTs’ prioritization of internal validity often comes at the expense of external validity (ie, generalizability to real-world clinical settings) [114]. Our review found that 14% (6/44) of studies prioritized external validity and evaluated their intervention via quasi-experimental methods. Further research in this area can provide valuable insights into real-world effectiveness and application of resilience and well-being DMHIs for MMs, PSP, and veterans. Given the high use of apps and programs, it is crucial to continue evaluating their efficacy and effectiveness, particularly prior to public use [32,115].

In addition, only 14% (6/44) of the studies were focused on feasibility or acceptability. Attention to these feasibility and acceptability issues is critical as these populations often do not engage with in-person mental health services [12,13] and experience a number of barriers to accessing services [13-17]. In addition, the high attrition rates, a notable limitation across the DMHI literature, emphasize the need for further investigation into acceptability, feasibility, and satisfaction with resilience and well-being DMHIs. For example, van Stolk-Cook et al [82] found that PTSD Family Coach 1.0 users opened their apps an average of 2.38 (week 1), 0.45 (week 2), 0.14 (week 3), and 0.22 (week 4) times across their study, and Parkes et al [83] found that participants used their app for a duration of 20.7 seconds (median). These examples are common throughout the DMHI research and provide valuable insights and a vital starting point for ongoing research.

A majority of the included research had participants use the app or program without additional support, but there were 9 interventions supplemented by clinician support, peer support, research staff support, mental health specialist support, feedback, or clinical monitoring [23,71-77]. There were no significant differences between groups on outcome measures across the 5 studies that compared clinician support versus no support [75], with or without treatment-as-usual [71-73], check-in versus self-managed groups [77], and peer support versus self-managed groups [76]. The only exception, however, is that Possemato et al [75] reported that those in the clinician-supported group were more likely to attend an additional PTSD session and accept a treatment referral. Therefore, these studies provide evidence that DMHIs may be just as effective without support; however, this may not be the case when compared with other treatment modalities. For example, Liu et al [32] found that self-guided resilience interventions appear to have little or no effect on increasing resilience, whereas there is a small, meaningful effect for in-person, remote, individual, and group interventions. Therefore, further research is needed to evaluate the effectiveness and efficacy of DMHIs compared with additional support and control conditions.

A potential weakness is that this literature is in its infancy for evaluating potential factors impacting intervention effectiveness. Many of these evaluations had small sample sizes and high attrition rates, posing challenges for detecting true mediation or moderation [116,117] and limiting both the internal and external validity of the results [118]. Although this was the case for many studies, 4 authors were able to successfully evaluate and find evidence for moderator variables, including parent gender [61], PTSD symptoms [79], combat exposure [79], self-efficacy [65], and interpersonal problems [80]. One study reported three significant mediators, including perceived norms of same-age peers (1) number of drinking occasions, (2) number of drinks, (3) and number of drinking occasions [81]. These variables, along with other potential moderators and mediators, should continue to be evaluated. This is an essential step in intervention research as it is necessary to explore potential factors that underlie or influence the association between an intervention and a desired outcome [119].

### Implications and Key Takeaways

This scoping review aimed to look at the breadth of literature for accessible and available DMHIs for MMs, PSP, and veterans, who are individuals who are regularly underserved and often do not access or have access to formal support. A key gap illuminated in this review is the lack of DMHIs developed and validated for MMs and PSP populations specifically. In addition, the vast majority of DMHIs focused on psychological well-being, while only a few focused on community, economic, and occupational well-being: important domains for one’s overall health, well-being, and resilience. Since 2020, there has been an increase in published literature on DMHIs for these populations; however, many DMHIs are available to these underserved populations before they have been empirically validated. For example, of the 44 papers included in this review, 13 were examined via RCT, and 4 via quasi-experimental design; therefore, additional evaluations of DMHIs for MMs, PSP, and veteran populations must be completed to examine the internal and external validity of these interventions.

There were 8 DMHIs for these populations that showed positive results via RCT and, therefore, may be recommended for these populations, including Concussion Coach, Family Foundations, Mission Reconnect, Resilience@Work, Support Coach, Thinking Forward, VetChange, and Virtual Hope Box. Although in clinical practice, DMHIs may be recommended as adjunct support between sessions, based on this review, it appears that some DMHIs are supported regardless of additional support, including PTSD Coach, Virtual Hope Box, and Thinking Forward. An additional consideration for future research and DMHI development includes the moderators that were found to have a significant impact on MMs, PSP, and veteran outcomes, such as baseline PTSD symptoms, combat exposure, self-efficacy, and interpersonal problems, as these factors may influence the effectiveness of the DMHI.

### Limitations and Strengths of the Review

Some methodological limitations exist in the current scoping review. Only English studies were included, and a gray literature search and snowball sampling were not used, potentially impacting the scope of this review. A potential limitation across this literature is defining *web-based programs*. Efforts by the research team to differentiate web-based programs from web-based courses, module-based courses or programs, chat rooms, Facebook groups, YouTube channels, and resource banks lead to determination of web-based programs as *interactive online platforms* that do not involve an invitation or log-in code provided by the DMHI developer. It would be beneficial, in the future, to have a clear consensus on the definition and further differentiate the aforementioned web-based resources.

This scoping review also has several strengths. The project used the PRISMA-ScR to ensure the study’s quality and minimize potential bias and included all search information to facilitate replication. In addition, a detailed, rigorous, and extensive search was conducted of 6 databases with additional search terms expanding upon previous reviews. At least 2 independent reviewers were engaged in the review, minimizing risk of bias. The study’s inclusion and exclusion criteria were established upfront and refined throughout. The scoping review also contributes to the literature by summarizing key information surrounding the quality of the evidence base for DMHIs for MMs, PSP, and veterans. This type of information is paramount as it is key to informing these populations and clinicians in terms of the best available DMHIs based on the empirical evidence, which is key to evidence-based practice and clinical decision-making [92]. This type of review and information is also key to providing app developers and researchers with information for next steps in terms of development, implementation, and research. Finally, the current project applied a whole-person approach by evaluating programs through the I-COPPE model [58] and cascading resilience framework [37]. These frameworks prioritize multidimensional and systems-based understanding of one’s well-being, allowing researchers and clinicians to focus on protective networks, rather than deficit-based frameworks.

Future research can build on the current project. First, it is important for future research to review DMHIs that involve synchronous or professional, peer, or researcher support. Such research may glean important information about the efficacy or effectiveness of resilience and well-being with guidance and support. In addition, it may clarify whether independent use of DMHIs is comparable with supported use of DMHIs. Second, future research should consider the acceptability of DMHIs across these populations. Although the current review grouped these populations (MMs, PSP, and veterans), we cannot speak to whether a DMHI developed for one population generalizes or is as effective for the other populations. Therefore, further research is needed to explore the acceptability of interventions across these populations and to explore ways in which DMHIs can be contextualized appropriately for each population.

### Conclusions

DMHIs have the potential to promote resilience and well-being in PSP, MMs, and veterans. This scoping review summarizes the state of the literature and surveys available DMHI programs and apps. Several themes related to DMHI literature were identified, together with intervention characteristics including the target populations, purpose, therapeutic approach, targeted symptoms, therapeutic modalities, evidence and methodologies used, and domains of well-being supported by identified programs and apps. More rigorous research is needed, however, to examine the effectiveness and efficacy, acceptability, feasibility, and satisfaction of DMHIs for MMs, PSP, and veterans. While the determination of DMHIs as an evidence-based alternative to in-person mental health care requires more research, the accessibility, customization, and scalability of DMHIs make this mode of delivery promising.

## Supplementary material

10.2196/65149 Multimedia Appendix 1 Search string example.

10.2196/65149 Multimedia Appendix 2 App and web-based program therapeutic approaches and modes of delivery.

10.2196/65149 Multimedia Appendix 3 Well-being domains supported by each program.

10.2196/65149 Multimedia Appendix 4 Summary of app and web-based program results.

10.2196/65149 Checklist 1 PRISMA-ScR checklist.

## Abbreviations

DMHI: digital mental health intervention
I-COPPE: interpersonal, community, occupational, physical, psychological, economic, and overall well-being
MM: military member
PPTE: potentially psychologically traumatic event
PRISMA: Preferred Reporting Items for Systematic Reviews and Meta-Analyses
PRISMA-ScR: Preferred Reporting Items for Systematic Reviews and Meta-Analyses extension for Scoping Reviews
PSP: public safety personnel
PTSD: posttraumatic stress disorder
RCT: randomized controlled trial
