# Supplementary material for: Digital Health Interventions for Military Members, Veterans, and Public Safety Personnel: Scoping Review
Source: JMIR Mhealth Uhealth. 2025 Oct 28;13:e65149. doi: 10.2196/65149 (PMC12560963; doi:10.2196/65149)
Supplement: Multimedia Appendix 1 [file mhealth-v13-e65149-s001.docx]

**Multimedia Appendix 1.**

**Example of Ovid Search**

Concept 1:

(Public Safety Personnel OR police* OR firefighter* OR **first responder*** OR firem* OR military OR soldier* OR army OR special force* OR (active AND duty) OR paramilitary OR armed-force* OR armed-service* OR servicewom* OR servicem*n OR air-personnel OR defence-force* OR **defense-force*** OR service-personnel OR navy OR air-force OR infantryman OR civil defence OR **civil defense** OR medic OR enlisted personnel OR reserve personnel OR **Royal Canadian Mounted Police** OR RCMP OR officer* OR **Emergency Medical Technician*** OR EMT OR **Emergency Medical Service*** OR EMS OR paramedic* OR **veteran*** OR **VA**):ti, ab

Concept 2:

(resilien* OR coping OR hardiness OR grit* OR **flourishing** OR **wellbeing** OR **well-being** OR **emotion regulation** OR **affect** OR (**self-manag*** AND **symptom***) OR **mood**): ti, ab

Concept 3:

(**virtual game** OR **online game** OR gaming OR **virtual play** OR **online play** OR videogam* OR **smart phone** OR **mobile app*** OR ((**Internet based**) AND (**intervention** OR **program**)) OR ((**online**) AND (**intervention** OR **program**)) OR ((**web based**) AND (**intervention** OR **program**)) OR **mental health app*** OR **smartphone app*** OR **smartphone-based** OR **smartphone application** OR **computerized** OR **computer-based** OR **iPAD** OR **computer based** OR **Internet format**): ti, ab

Limits: English articles from 2000 onwards

Search fields: Titles and Abstracts

Search Mode: Boolean

Results: 174 search hits

*Note:* Non-bolded items were from Voth et al.’s (2020) original scoping review, and bolded terms were added to our current review.
