# Supplementary material for: Digital Health Interventions for Military Members, Veterans, and Public Safety Personnel: Scoping Review
Source: JMIR Mhealth Uhealth. 2025 Oct 28;13:e65149. doi: 10.2196/65149 (PMC12560963; doi:10.2196/65149)
Supplement: Multimedia Appendix 2 [file mhealth-v13-e65149-s002.docx]

**Multimedia Appendix 2.**

**Table S1.** Summary of program characteristics.

| Author | DMH Intervention(s) | Year of Publication | Location of Study | Therapeutic Orientation | Available as an app, online, or both |
| --- | --- | --- | --- | --- | --- |
| Acosta et al [69] | Thinking Forward | 2017 | USA | CBT*Strengths-based*Self-management | Online |
| Albright et al [64] | Family of Heroes | 2012 | USA | Social cognitive theory | Online |
| Babson et al [84] | CBT-Insomnia Coach | 2015 | USA | CBT | App |
| Belanger et al [65] | Concussion Coach | 2022 | USA | Unspecified | App |
| Brief et al [70] | VetChange | 2013 | USA | CBT*MI*Self-control training | Both |
| Brief et al [79] | VetChange | 2018 | USA | CBT*MI*Self-control training | Both |
| Buck et al [78] | FOCUS | 2022 | USA | CBT*Behavioural activation*Psychoeducation*Social skills | App |
| Bush et al [71] | Virtual Hope Box | 2017 | USA | CBT*Dialectical Behavioral Therapy*Emotion regulation | App |
| Bush et al [74] | Virtual Hope Box | 2015 | USA | CBT*Dialectical Behavioral Therapy*Emotion regulation | App |
| Bush et al [85] | AfterDeployment.org | 2014 | USA | Cognitive Behavioral Therapy (CBT)*Acceptance and Commitment Therapy (ACT)*Stress inoculation | Online |
| Bush et al [72] | T2 Mood Tracker | 2014 | USA | Self-monitoring*Patient-Centered | App |
| Chen et al [86] | Virtual Hope Box | 2018 | USA | CBT*Dialectical Behavioral Therapy*Emotion regulation | App |
| Coifman et al [87] | Daily Coping Toolkit | 2021 | USA | Unspecified | App |
| Davis et al [88] | Mind Guide | 2023 | USA | Mindfulness | App |
| Denneson et al [73] | Virtual Hope Box | 2019 | USA | CBT*Dialectical Behavioral Therapy*Emotion regulation | App |
| Dillon et al [89] | Mobile Anger Reduction Intervention | 2023 | USA | Interpretation bias modification | App |
| Engel et al [90] | Delivery of Self Training and Education for Stressful Situations Primary care version | 2015 | USA | CBT | Online |
| Feinberg et al [61] | Family Foundations | 2020 | USA | Unspecified | Online |
| Heyen et al [91] | COVID-19 Anxiety and Stress Resilience Training | 2021 | Switzerland | CBT | Online |
| Higgins et al [92] | Pain eHealth for Activity, Skills, and Education | 2020 | USA | CBT for Chronic Pain*Self-management*SMART Goals | Online |
| Hofmann et al [93] | Coping with Suicide Prevention | 2021 | Germany and Switzerland | CBT*Psychoeducation | Online |
| Ivory [94] | Curable | 2023 | USA | Psychoeducation*CBT | App |
| Johnson et al [95] | Health eRide: Your Journey to Managing Pain | 2017 | USA | MI | Online |
| Joyce et al [67] | Resilience@Work | 2019 | Australia | Mindfulness*ACT* Compassion-Focused Therapy | Online |
| Joyce et al [96] | Resilience@Work | 2018 | Australia | Mindfulness*ACT* Compassion-Focused Therapy | Online |
| Kahn et al [66] | Mission Reconnect | 2016 | USA | Biopsychosocial model*Mindfulness*Massage Therapy*Psychoeducation*Positive emotions | Online |
| Kuhn et al [97] | Insomnia Coach | 2022 | USA | CBT*Sleep restriction therapy*Stimulus control* Relaxation*Psychoeducation | App |
| Kuhn et al [98] | PTSD Coach | 2014 | USA | CBT*Self-control training | App |
| Leightley et al [23] | Information about Drinking for Ex-serving personnel | 2018 | United Kingdom | Self-monitoring*Applied behavior change | Online |
| Livingston et al [99] | VetChange | 2020 | USA | CBT*MI*Self-control training | Both |
| McLean et al [77] | Renew | 2022 | USA | Self-management*Imaginal exposure*Psychoeducation | App |
| Newberger et al [100] | VetChange | 2023 | USA | CBT*MI*Self-control training | Both |
| Parkes et al [83] | Mental Health Toolkit for Veterans Project | 2023 | United Kingdom | CBT | App |
| Polizzi et al [80] | VetChange | 2024 | USA | CBT*MI*Self-control training | Both |
| Possemato et al [101] | Coming Home and Moving Forward (now called Thinking Forward) | 2015 | USA | CBT*Strengths-based*Self-management | Online |
| Possemato et al [75] | PTSD Coach | 2016 | USA | CBT*Self-control training | App |
| Possemato et al [76] | Thinking Forward | 2019 | USA | CBT*Strengths-based*Self-management | Online |
| Roy et al [102] | a. LifeArmor,  b. Prolonged exposure Coach,  c. Positive Activity Jackpot,  d. Eventful,  e. Tactical Breather,  f. Virtual Hope Box,  g. Daily Yoga, or  h. Simply Yoga | 2017 | USA | a. Psychoeducation  b. Exposure Therapy*Psychoeducation  c. Social Engagement  d. Social Engagement  e. Relaxation*Stress management  f. CBT*Dialectical Behavioral Therapy*Emotion regulation  g. Relaxation  h. Relaxation | a. App  b. App  c. App  d. App  e. App  f. App  g. App  h. App |
| Solar et al [103] | Pain eHealth for Activity, Skills, and Education | 2021 | USA | CBT for Chronic Pain*Self-management*SMART Goals | Online |
| van der Meer et al [68] | Support Coach | 2020 | Netherlands | CBT*Psychoeducation*Social support | App |
| van Stolk-Cooke et al [82] | PTSD Family Coach 1 | 2023 | USA | Mindfulness*Stress management*relationship/communication skills | App |
| Vaughan et al [62] | Building Resilience | 2020 | Canada | Unspecified | Online |
| Wild et al [45] | Mind Online | 2016 | England | Mindfulness*Psychoeducation*Stress management | Online |
| Williams et al [81] | Alcohol Savvy | 2009 | USA | Motivational Interviewing (MI)*Social learning theory | Online |
| Williams et al [81] | Drinker's Check-Up | 2009 | USA | MI*Social learning theory | Online |
