# Supplementary material for: Digital Health Interventions for Military Members, Veterans, and Public Safety Personnel: Scoping Review
Source: JMIR Mhealth Uhealth. 2025 Oct 28;13:e65149. doi: 10.2196/65149 (PMC12560963; doi:10.2196/65149)
Supplement: Multimedia Appendix 3 [file mhealth-v13-e65149-s003.docx]

**Multimedia Appendix 3.**

**Table S1.** I-COPPE domains supported by each program.

| App Name | Interpersonal | Community | Occupational | Psychological | Physical | Economic | Overall |
| --- | --- | --- | --- | --- | --- | --- | --- |
| After-deployment |  |  |  | ✓ |  |  |  |
| Building Resilience |  |  |  | ✓ |  |  |  |
| CBT-Insomnia Coach |  |  |  | ✓ | ✓ |  |  |
| Coming Home and Moving Forward |  |  |  | ✓ | ✓ |  |  |
| Concussion Coach |  |  |  | ✓ | ✓ |  |  |
| Coping with Suicide-Prevention |  | ✓ | ✓ | ✓ |  |  |  |
| COVID-19 Anxiety and Stress Resilience Training |  |  |  | ✓ |  |  |  |
| Curable |  |  |  | ✓ | ✓ |  |  |
| Daily Coping Toolkit |  |  |  | ✓ |  |  |  |
| Delivery of Self Training and Education for Stressful Situations Primary care version |  |  |  | ✓ |  |  | ✓ |
| Drinker’s Check-up and Alcohol Savvy |  |  |  | ✓ | ✓ |  |  |
| Family Foundations | ✓ |  |  | ✓ |  |  |  |
| Family of Heroes | ✓ | ✓ |  |  |  |  |  |
| FOCUS | ✓ |  |  | ✓ |  |  |  |
| Health eRide: Your Journey to Managing Pain | ✓ |  |  | ✓ | ✓ |  |  |
| Information about Drinking for Ex-serving personnel |  |  |  | ✓ | ✓ |  |  |
| Insomnia Coach |  |  |  | ✓ | ✓ |  |  |
| LifeArmor, Prolonged Exposure Coach, Positive Activity Jackpot, Eventful, Tactical Breather, Virtual Hope Box, Daily Yoga, and Simply Yoga | ✓ |  |  | ✓ | ✓ |  |  |
| Mental Health Toolkit for Veterans Project (MeT4VeT) |  |  |  | ✓ |  |  |  |
| Mind Guide |  |  |  | ✓ | ✓ |  |  |
| Mind Resilience Intervention | ✓ |  |  | ✓ |  |  |  |
| Mission Reconnect | ✓ |  |  | ✓ | ✓ |  |  |
| Mobile Anger Reduction Intervention |  |  |  | ✓ |  |  |  |
| Pain eHealth for Activity, Skills, and Education | ✓ |  |  | ✓ | ✓ |  |  |
| PTSD Coach | ✓ |  |  | ✓ |  |  |  |
| PTSD Family Coach 1 | ✓ |  |  | ✓ |  |  |  |
| Renew | ✓ |  |  | ✓ | ✓ |  |  |
| Resilience@Work |  |  |  | ✓ |  |  |  |
| Support Coach | ✓ |  |  | ✓ |  |  |  |
| T2 Mood Tracker |  |  |  | ✓ |  |  |  |
| Thinking Forward | ✓ |  |  | ✓ | ✓ |  | ✓ |
| VetChange | ✓ |  |  | ✓ | ✓ |  |  |
| Virtual Hope Box | ✓ |  |  | ✓ |  |  |  |
