# Supplementary material for: Digital Health Interventions for Military Members, Veterans, and Public Safety Personnel: Scoping Review
Source: JMIR Mhealth Uhealth. 2025 Oct 28;13:e65149. doi: 10.2196/65149 (PMC12560963; doi:10.2196/65149)
Supplement: Multimedia Appendix 4 [file mhealth-v13-e65149-s004.docx]

**Multimedia Appendix 4.**

**Table S1.** Summary of program results sorted by I-COPPE domain.

| **I-COPPE Dimension** | | | | | | | | | | | |
| --- | --- | --- | --- | --- | --- | --- | --- | --- | --- | --- | --- |
| Program Name | Length of Intervention | Research Design | | Participant description: Total *N* (group of interest *n*) | | Attrition Rates | | | Results (significant*) | |  |
| **Community and Interpersonal** | | | | | | | | | | | |
| Family of Heroes^a^ [64] | 1 hour | RCT: Control versus experimental | | Family members of veterans:  *N* = 94 (Family of Heroes *n* = 50) | | 0% | | | Family members felt more prepared to recognize signs* and voice concerns* when their veteran was experiencing post-deployment stress, and felt more prepared to motivate* them to seek help at the VA. | |  |
| **Community, Occupational, and Psychological** | | | | | | | | | | | |
| Coping with Suicide (COPS) Prevention^b^ [93] | 3 weeks | Quasi-  experimental design | | PSP (police):  *N* = 142 | | Post-training:  28%    Follow-up:  82% | | | Improvement in their self-assessed competence in delivering death notifications*, in suicide prevention*, and in dealing with one’s own mental health.* Self-assessed knowledge* improved, specifically, knowledge for delivery death notifications*, for suicide prevention*, and for mental health.* | |  |
| **Interpersonal and Psychological** | | | | | | | | | | | |
| Family Foundations^b^ [61] | 6 months | Pilot RCT: Family Foundations versus control | | MM heterosexual couples who were expecting their first child, living together and one partner was in the military  *N* = 56  (*n* = 29 FF) | | Controls:  Mothers (7.4%)  Fathers (22.2%)    Intervention:  Mothers (34.5%)  Fathers (48.3%) | | | Intervention couples completed an average of 3.93 of the 8 modules. For depression, intervention parents reported lower levels of depression.* Intervention mothers reported significantly higher levels of co-parenting closeness* and support* compared with control mothers. In the intervention group, parents reported that their children demonstrated significantly lower levels of sadness.*  Intervention condition mothers reported their infants as higher on self-soothing than control mothers.* Dosage effects for intervention couples (completing more than four modules vs. four or fewer) for conflict resolution style* and co-parenting support.* There was a dosage effect for father-reported parenting undermining* but not mothers. | |  |
|  |  |  | |  | |  | | |  | |  |
| FOCUS^a,b^ [78] | 4 weeks | Pilot Study: Quasi-  experimental, feasibility, and acceptability | | Veterans with psychosis:  *N* = 17 | | 6% | | | Most participants selected that they felt satisfied and confident/comfortable using the app. Most would recommend the program. They reported some improvements in quality of life, recovery, and severity of voices. | |  |
| Mind Online^b^ [45] | 6 weeks | RCT: Mind resilience group intervention versus mind online psychoeducation | | PSP (police, fire, ambulance, search-and-  rescue):  *N* = 317 (mind online *n* =113) | | Resilience intervention:  Post 19%  Follow-up 11%    Online psychoeducation:  Post 19%  Follow-up 12% | | | Mind online psychoeducation group completed more modules than resilience groups*, but the resilience group rated the intervention as more helpful.* Small non-significant between- and within-group effect sizes suggest there was little to no change on these measures resulting from either intervention. | |  |
| PTSD Coach [98] | 3 days | Pilot Study: User satisfaction, perceived helpfulness, and usage patterns | | Veterans attending PTSD treatment programs:  *N* = 45 | | 0% | | | Overall feedback suggests that PTSD Coach was helpful for managing acute stress, reducing stress, reducing PTSD symptoms, and facilitating conversations about PTSD with friends and family. Participants felt PTSD Coach was convenient and simple to use, easy to carry, and readily accessible at any time. | |  |
| PTSD Coach [75] | 8 weeks | Pilot RCT: App use with versus without clinician support | | Veterans (primary care patients who screened positive for PTSD)  *N* = 20 (self-managed  *n* = 10) | | Clinician supported: 0%    Self-managed: 10% | | | Both groups experience a decline in PTSD symptoms pre to post*, with no difference between groups. Clinician supported group was more likely to accept a referral for mental health treatment* and to attend at least one additional session focused on PTSD* compared to the self-managed group. | |  |
| PTSD Family Coach [82] | 4 weeks | Pilot Study: RCT, feasibility, and acceptability | | Family member of vet with PTSD: *N* = 200 (PTSD family coach *n* = 104; psychoeducation app (active control) *n* = 96) | | 49.5% did not use app at least once in 4 weeks  40% did not complete post-treatment survey | | | PTSD Family Coach 1.0 users opened their apps an average of 2.38 (week 1), 0.45 (week 2), 0.14 (week 3), and 0.22 (week 4) times. Participants rated the app a mean of 4.77 (out of 8) on app satisfaction, and 2.99 (out of 5) on perceived helpfulness. No significant difference between user satisfaction or helpfulness by condition, and no significant treatment by time interaction effects between conditions. No significant correlations between the number of times the app was opened and changes in outcomes of interest for either condition. Post hoc analysis collapsing conditions to compare app users (n=101) with app nonusers (n=99) revealed no significant differences between groups for any demographic variables or outcome variables of interest at baseline. There were treatment-by-time interaction effects for changes in perceived stress*, such that app users experienced reductions in perceived stress, while app nonusers did not. No other significant treatment-by-time interaction effects were identified. | |  |
| Support Coach [68] | 1 month | RCT: Support Coach versus control | | PSP (nurses, physicians, paramedics, ambulance drivers)  *N* = 287 (Support Coach *n* = 143) | | Support Coach:  44.7%    Control  27.7% | | | Initial elevated PTSD symptoms*, elevated negative trauma-related cognitions*, and lower psychological resilience* related to more Support Coach exercises performed. Both groups showed a reduction in PTSD symptoms* (no group difference). Improvements for Support Coach in terms of negative cognitions*, resilience*, and perceived lack of social support*, with negative cognition* and resilience* change differing from controls. | |  |
| Virtual Hope Box (VHB) [74] | 6-8 weeks | Prototype design and clinical proof-of-concept testing: cross-over, counterbalanced design (order of use randomized VHB vs. Conventional Hope Box [CHB]) | | Veterans with high-risk-of-  self-harm enrolled in DBT:  *N* = 18 | | 0% | | | Participants preferred and used VHB more, but most participants said they would recommend both VHB and CHB combined. Participants found the app easy to use and felt they would use it in the future and recommend it to others. Participants reported that it helped manage distress, negativity, hopelessness, and anger. | |  |
|  |  |  | |  | |  | | |  | |  |
|  |  |  | |  | |  | | |  | |  |
| VHB [71] | 12 weeks | RCT: TAU versus TAU + VHB | | Veterans in active treatment for suicidal ideation:  *N* = 118 (VHB + TAU *n* = 58) | | 9% | | | VHB showed improvement in enlisting support from family and friends.* VHB group showed greater ability compared to TAU to cope with unpleasant emotions/thoughts*, and greater odds of identifying the treatment as helpful* and likely to use it again.* | |  |
| VHB [86] | 12 weeks | Secondary analysis: Bush et al. ^11^ RCT focused only on TAU + VHB | | Veterans with risk of suicide:  *N* = 58 | | 14% | | | Participants had a difficult time identifying supportive contacts to add to VHB. Difference between low app use and high app use groups for improvements in self-efficacy.* Lower usage was associated with greater ability to stop negative thoughts*, and higher usage was associated with improvements in reasons for living.* | |  |
| VHB [73] | 12 weeks | Secondary analysis: Bush et al. ^11^ RCT TAU versus TAU + VHB | | Veterans with suicidality in active treatment  *N* = 117 (VHB + TAU *n* = 58) | | VHB + TAU:  15.5%    TAU:  6.77% | | | VHB groups showed small increases in self-efficacy and small decrease in suicidal ideation. There were no discernible differences between treatment groups. No indirect effect of treatment on suicidal ideation through coping self-efficacy. | |  |
| **Interpersonal, Physical, and Psychological** | | | | | | | | | |  |  |
| Health eRide: Your Journey to Managing Pain^b^ [95] | 4 weeks | Pilot Study: Quasi-  Experimental design | | Veterans with chronic pain:  *N* = 69 | | 36.23% | | | Overall reductions in PTSD symptoms* and levels of pain (pain now*, usual pain*, best pain*, worst pain*, and pain impact*). Improvements in pain coping skills, such as exercise*, relaxation*, cognitive control*, and use of proper body mechanics.* 68% reported a slight to considerable improvement, and 32% stating no noticeable change or no difference in their condition. 95% liked the information and content, and felt it was easy to use. 57% reported they disliked the length of the program, were confused about the design and questions, and felt the program did not provide new information. | |  |
|  |  |  | |  | |  | | |  | |  |
| LifeArmor^b^,  Prolonged exposure (PE) Coach,  Positive Activity Jackpot^b^,  Eventful,  Tactical Breather^b^,  Virtual Hope Box,  Daily Yoga^a^, or  Simply Yoga^a^ [102] | 6 weeks | RCT: Resilience enhancement group (RE) versus control group (CT) | | MM and Veterans in the military healthcare system that experienced a stressful event:  *N* = 144 (CT is the focus of this review: *n* = 72; *n* = 30 for follow-up). | | RE Group:  17.2%    CT Group:  3.33% | | | RE participants demonstrated greater overall app use compared to CT*. RE participants used LifeArmor*, PE Coach*, and Tactical Breather*, more often than CT. RE identified LifeArmor* and PE Coach* as most useful compared to CT, and CT more frequently identified meditation* and yoga* apps. From baseline, both groups showed reductions in PTSD symptoms*, re-experiencing*, and depression* at 6-weeks and 3-months, with no significant group differences. Similarly, both groups showed reductions in avoidance* and hyperarousal* at 3-months, with no significant group differences. Concussion with loss of consciousness was associated with higher 6-week depression* and anxiety*, and with higher 3-month PTSD symptoms.* Both groups showed reductions in PTSD symptoms and re-experiencing baseline to 6 months*, and 12 months* follow-up. There were no group differences at 3-, 6-, 12-month follow-up. | |  |
| Mission Reconnect (MR)^a^ [66] | 16 weeks | RCT: 4-arm to evaluate both comparative and additive effects: (1) MR alone, (2) MR+PREP, (3) PREP alone, and (4) waitlist control | | Veterans and partners:  *N* = 181 (veterans); *n* = 160 (dyads) | | Arm 1: 1%    Arm 2: 4%    Arm 3: 4%    Arm 4: 1% | | | MR showed general improvements in perceived stress*, depression*, PTSD symptoms*, self-compassion*, response to stressful experiences*, and sleep quality* (only T1-T2). At T1-T2, MR+PREP showed reductions in depression*, and T1-T3 showed improvements in self-compassion*. For between group comparisons of the four treatment arms, MR was favored in terms of improvements in usual pain in past week* (compared to MR+PREP), response to stressful experiences* (compared to PREP), and perceived stress*, self-compassion*, usual pain in past week* (compared to waitlist). MR+PREP showed improvements in adjustment* compared to PREP and to the waitlist. The massage feature was helpful immediately post-massage for both veterans and their partners in terms of rated reductions in physical pain*, tension*, irritability*, anxiety*, and depression*. Over 8-weeks, as related to the massage feature, veterans reported reductions in physical tension*, irritability* and anxiety*, and partners reported reductions in anxiety*. | |  |
| Pain eHealth for Activity, Skills, and Education (Pain EASE)^b^ [92] | 10 weeks | 2-Phase Design: (1) development, feedback, and program design, and  (2) feasibility and preliminary efficacy | | Veterans with chronic lower back pain:  *N* = 73 (Phase I: development of Pain EASE Prototype *n* = 15; Phase 2: feasibility *n* = 58) | | 29.3% | | | During phase 1, participants suggested website style changes, content reduction, the addition of “Test Your Knowledge” quizzes, and CBT skill practice self-monitoring form revisions, all of which were completed prior to the start of Phase II. On a measure of treatment satisfaction, only 40% of Phase II participants reported most or all their needs were met by the Pain EASE program. On average, participants logged on 6 times in 10 weeks and accessed less than four of the 10 modules. Results showed reduction in pain interferences*, depression*, tension*, and snoring*. | |  |
| Pain EASE [103] | 10 weeks | Secondary Analysis: Higgins et al. ^17^ correlational evaluation of predictors of app engagement | | Veterans with moderate to severe chronic pain coping scores and low back pain:  *N* = 58 | | 62% did not use more than 3 modules | | | Number of logins was correlated with all measures of engagement.* Completed weekly calls was associated with the number of logins*, skill modules accessed*, and completed quizzes.* Race* and age* predicted the number of skill modules accessed, such that White-identified and older participants were more likely to access modules. | |  |
| Renew [77] | 6 weeks | Pilot RCT: Waitlist versus intervention with support versus intervention without support | | Veterans:  *N* = 93 (with support *n* = 31; without support *n* = 31) | | Renew: 29%    Renew + coaching: 19.35%    Waitlist: 19% | | | Number of support persons added to the app was associated with greater time spent in app*, greater time spent in exposure*, and receiving more points associated with the app*. Intervention participants experienced decreased PTSD symptoms from baseline to posttreatment*, but not the delayed use group. During app use, the delayed use group experienced a decrease in PTSD symptoms*. No group difference in PTSD symptom reduction or engagement indices for the support condition compared to the no support condition. | |  |
| VetChange [70] | 8 weeks | RCT: intervention versus delayed intervention | | Veterans with Alcohol use and PTSD symptoms:  *N* = 592 (initial VetChange *n* = 404; delayed VetChange  *n* = 190) | | IIG:  54.7%    DIG:  60.2% | | | Intervention group compared to delayed intervention demonstrated reductions in drinks per drinking day*, average weekly drinks*, percent heavy drinking days*, and PTSD symptoms*. During wait period, the delayed intervention groups showed reductions in drinks per drinking day*, average weekly drinks*, percent heavy drinking days*, and PTSD symptoms*, but less so than the intervention group*. Intervention period for both groups led to reductions in drinks per drinking day*, average weekly drinks*, and PTSD symptoms*, however, only the initial intervention group showed reductions in percent heavy drinking days*. From end of intervention to 3-months follow-up, both groups showed reductions in drinks per drinking day*, average weekly drinks*, percent heavy drinking days*, and (only for the delayed intervention group) reductions in PTSD symptoms*. | |  |
| VetChange [79] | 8 weeks | Secondary analysis: Brief et al. ^20^ RCT intervention versus delayed intervention group | | Veterans with Alcohol use and PTSD symptoms:  *N* = 523 | | 50.3% | | | Those with heightened baseline PTSD symptoms at baseline showed a sharper decline in drinks per drinking day*, average weekly drinks*, and percent heavy drinking days* at the end of the intervention, with no difference in rate of change of alcohol use. Similarly, they showed a sharper decline from baseline to follow-up for alcohol-related problems*, but not for other measures of alcohol use. This moderation was not significant from the end of intervention to follow-up. Participants that reported higher levels of combat exposure at baseline showed sharper decline (baseline to end of intervention and end of intervention to follow-up) for average weekly drinks*, but did not at baseline to end of intervention nor for other alcohol use measures. | |  |
| VetChange [99] | 6 months | Quasi-  experimental design | | Veterans with hazardous alcohol use:  *N* = 222 | | Month 1: 52%  Month 2: 58%  Month 6: 74% | | | Veterans reported a significant reduction in average weekly drinks over time (0-6 months)*. Change in average weekly drinks in the first month was positively associated with only hyperarousal symptoms between 1 and 6 months*. After adjusting for time, there was an interaction between hyperarousal symptoms and time on dropout*, and a main effect of hypervigilance*. | |  |
| VetChange [80] | 8 weeks | Secondary analysis: Brief et al. ^20^ RCT intervention versus delayed intervention group | | Veterans with Alcohol use and PTSD symptoms:  *N* = 592 (initial VetChange *n* = 404; delayed VetChange  *n* = 190) | | IIG:  54.7%    DIG:  60.2% | | | Those that scored high on the Short Inventory Problems Interpersonal (SIP-I) scale exhibited higher interpersonal problems. The high SIP-I group showed greater PTSD symptoms at baseline compared to the med and low SIP-I groups*. Individuals endorsing greater interpersonal problems (i.e., high SIP-I group) demonstrated larger reductions in PTSD symptoms over time*, which was found only in the IIG group. | |  |
| **Interpersonal, Overall, Physical, and Psychological** | | | | | | | | | | | |
| Thinking Forward (TF)^b^ [69] | 12 weeks | RCT: TAU versus TAU + TF | | Veterans:  *N* = 162 (TAU + TF *n* = 81) | | 23.5% | | | Overall effect of time on changes in drinks per drinking day*, percent drinking days*, percent drug use days*, PTSD severity*, but not for quality of life both physical and psychological. Those that completed all modules showed no treatment by time differences. TF group reported greater declines compared to TAU for perceived heavy drinking days*, but no group differences for percent drug use days, PTSD symptoms, and quality of life. | |  |
| TF^b^ [76] | 12 weeks | Pilot RCT: with versus without peer support, and feasibility study | | Veterans with PTSD (or subclinical) and hazardous alcohol use:  *N* = 30 (self-managed is the focus of this review *n* = 15) | | 7% | | | 7/9 veterans were very satisfied and felt TF helped them deal more effectively with problems. No significant difference between groups for alcohol use measures, PTSD symptoms, resilience, social quality of life, coping, and psychological quality of life. Significant change in peer support group for PTSD symptoms (Confidence interval (CI) includes zero)*, coping*, resiliency (note, CI includes zero)*, social quality of life (note, CI includes zero)*, psychological quality of life*. High activation (taking action and staying the course under stress) participants reported a larger decrease in PTSD severity* and reduction in drinking days per month* compared to low activation participants (person sees value in taking an active role)*, which was consistent in both groups. | |  |
| VetChange [100] | 6 months | Secondary Analysis: Livingston et al. ^22^ quasi-experimental design | | Veterans with hazardous drinking:  *N* = 222 | | 1-month 52%  3-month 58%  6-month 74% | | | Note: this evaluation of VetChange was included in this I-COPPE domain given that satisfaction with life was included as an outcome measure (an indicator of overall well-being) and because the authors believed the app would improve overall well-being.  Same results/population as Livingston et al. ^22^ in terms of drinking reduction. Satisfaction with life increase over 6 months of app use* but was not related to the greater reduction in drinking in the first month. Reductions in PTSD symptom severity by 1-month predicted improvement in satisfaction with life over the six months. | |  |
| **Overall and Psychological** | | | | | | | | | | | |
| Delivery of Self Training and Education for Stressful Situations Primary care version (DESTRESS-PC)^b^ [90] | 6-weeks | RCT: DESTRESS-PC versus optimized usual PTSD care (OUC) | | MM and veterans with diagnosed PTSD:  *N* = 80 (OUC + DESTRESS-PC *n* = 43) | | 17.5% | | | DESTRESS-PC group improved at a faster rate than the OUC group and showed larger treatment gains for treatment by time interaction* at 6-, 12-*, and 16-weeks. For depression symptoms, there was a main effect for time.* No significant interaction (treatment by time) for depression, somatic symptoms, and mental and physical quality of life. Positive correlation between the number of logins completed and the decrease in PTSD symptoms at 12-weeks.* | |  |
| **Physical and Psychological** | | | | | | | | | | | |
| Alcohol Savvy (AS)^b^ [81] | 6 months | RCT: AS versus DCU versus control | | MM  *N* = 3070 (DCU *n* = 1470; AS *n* = 686) | | 1-month 55%  6-month 77% | | | No app direct effects at 1 and 6 months. At one month follow-up there was one mediator identified. As exposure increased perceived norms of number of drinking occasions for same-age peers* increased average number of days that alcohol was consumed* and decreased average number of drinks consumed per drinking occasion*. | |  |
| Drinker's Check-Up (DCU)^a,b^ [81] |  |  | |  | |  | | | At 1-month follow-up direct app effects for average number of days alcohol was consumed, binge drinker status, heavy drinker status, estimated peak BAC, drinks consumed per drinking occasion, and number of days perceived drunk. At one month follow-up there were two mediators identified. First, DCU decreased the perceived quantity of same-age peer number of drinks* which increased number of days perceived drunk*, increased binge drinking episodes*, increased heavy drinker status*, increased estimated peak BAC*, and increased average number of drinks consumed per drinking occasion*. Second, DCU exposure decreased perceived norms of number of drinking occasions for same-age peers*, which increased the number of days that alcohol was used* and decreased perceived norms of number of drinking occasions for same-age peers*. At six months, the app decreased the number of days alcohol was consumed and drinks consumed per drinking occasion. At six-month follow-up there was one mediator identified. DCU exposure decreased perceptions of same-age peers’ number of drinks* which then increased binge drinking episodes*, increased heavy drinker status, increased binge drinker status*, and increased average number of drinks consumed per drinking occasion*. | |  |
| CBT-Insomnia Coach (CBT-I) [84] | 2 weeks | Pilot RCT: placebo vs treatment, and qualitative feedback | | Veterans with sleep problems and cannabis use:  *N* = 4 (CBT-I n = 2) | | CBT-I: 0%  Controls: 25% | | | CBT-I group reported greater engagement and felt the app was helpful, easy to use and accessible. Controls dropped out or used the app minimally. | |  |
| Concussion Coach [65] | 12 weeks | RCT: Concussion Coach versus TAU | | Veterans with mild traumatic brain injury post-concussive symptoms:  *N* = 479 (Concussion Coach *n* = 238) | | 19.3% | | | The Concussion Coach group had 29% greater odds of improvements in post-concussive symptoms than TAU.* Veterans who engaged in Concussion Coach were 36% decrease in the odds of reporting worsening post-concussive symptoms* and a 25% decrease in the odds of reporting increased psychological distress. For the Concussion Coach group, there was a 42% greater probability of increased self-efficacy* compared to TAU. When self-efficacy was increased, there was a greater probability of reduction in PTSD symptoms* and psychological distress.* Time on Concussion Coach negatively correlated with post-concussive symptom severity change.* Post-concussive symptom severity change positively correlated with psychological distress change* and negatively correlated with efficacy change.* Psychological distress change negatively correlated with efficacy change.* | |  |
| Coming Home and Moving Forward^b^ [101] | Participants presented with outlines | Qualitative: focus groups (veterans); individual interviews (veterans); and expert feedback (delphi procedure) | | Veterans  *N* = 18 (focus group); *n* = 34 (individual feedback) | | 0% | | | Part 1 expert feedback consisted of strategies to execute the content or of examples to increase credibility and buy-in.  Part 2 veteran focus group suggested the program would be appropriate for people that have been back from deployment for several months. Three themes emerged to consider or add to the program: (1) use of technology and privacy, (2) use of strengths-based approach, and (3) adding desired module content. Part 3 was individual veteran feedback. 6 modules were adapted or were retained and made optional or mandatory based on this feedback. Participants enjoyed that it was simple, useful, informative and offered a unique way to address problems. Participants disliked clicking too much to get through one module, quiz questions presenting more than once, or that they could not relate to the automatic thoughts or drugs/alcohol modules. Final programs consisted of 24 CBT skill modules that are self-paced and take 15-25 minutes to complete. | |  |
| Curable [94] | 30 days | Quasi-  experimental design | | Veterans with chronic pain  (*N* = 8) | | 12.5% | | | Out of eight participants, seven experienced a reduction in chronic pain scores, seven experienced a decrease in their pain sensations, six perceived a change in their pain severity and symptoms, and all reported a positive improvement. All eight participants said they would recommend Curable to other veterans with chronic pain. Seven reported improvement in the pain they are currently experiencing, four reported improved quality of life (with one decreasing), and six reported their quality of life improving as a result of the intervention. Six showed improvements in PTSD symptoms, and two remained the same. | |  |
| Information about Drinking for Ex-serving personnel (InDEx)^b^ [23] | 4 weeks | App development (Agile methodology) and quasi-  experimental design | | Veterans with hazardous alcohol use:  *N* = 31 | | Week 1: 19%  Week 2: 19%  Week 3: 32%  Week 4: 29% | | | Participants used the app for a median of 4 weeks, 15 times over 4 weeks**.** Binge drinking days per week remained similar at week 1 versus week 4. Units per drinking day and units consumed from week 1 to week 4 were reduced. AUDIT score (alcohol use) reduced slightly but still remained in the hazardous use range. | |  |
| Insomnia Coach [97] | 6 weeks | Pilot Study: RCT, feasibility, acceptability, and follow-up qualitative interview | | Veterans with insomnia symptoms:  *N* = 50 (Insomnia Coach *n* = 25 (interview *n* = 15)) | | Treatment:  Post-treatment 4%  12-weeks 16%    Waitlist:  Post-treatment 4%  12-weeks 12% | | | On average, participants used the app on 50.2% of days during the treatment period and 30.3% of days during the follow-up period. After the treatment period, there was a positive effect on sleep-related impairment*. There was a positive effect on sleep-related impairment*, insomnia severity index*, sleep onset latency*, sleep quality*, and depression*. During the follow-up, barriers reported were inability to adhere to sleep recommendations (n = 4), inability to commit to ongoing use of app (n = 2), and confusion with using app (n = 2). Participants reported changes in knowledge (n = 13), changes in sleep behaviours (n = 11), and changes in sleep efficiency (n = 13). Nearly all (n = 11) described Insomnia Coach in positive terms and would recommend it to a friend. | |  |
| Mind Guide [88] | 2 weeks | Phase 1: Intervention development  Phase 2: Beta testing  Phase 3: Pilot RCT protocol | | Phase 1: Subject matter experts;  Phase 2: Veterans with PTSD and AUD that are not currently seeking treatment:  *N* = 16 | | 0% | | | Phase 1: Content was developed by subject matter experts across six months of bi-weekly meetings to build upon Mindfulness Coach, including information about the triangle of awareness, and specific meditations for cravings.  Phase 2: Most participants reported Mind Guide as a positive experience and some felt it aided in their understanding of mindfulness. Some reported that Mind Guide aided them in dealing with symptoms of PTSD, and that the app was useful for tracking mindfulness cues and triggers, letting go of negative thoughts, recognizing negative self-talk, and improving self-compassion. Not all felt mindfulness supported their symptoms of PTSD or alcohol use. Participants requested the following changes: clearer and more tracking systems, ability to export their progress, more reminder notifications, clearer instructions for the guided portions of modules, shorter meditations, and testimonial videos. Overall, participants showed a reduction in frequency of alcohol use*, alcohol problems*, PTSD symptoms*, re-experiencing*, avoidance*, negative alterations in mood*, hyperarousal*, expressive suppression*, craving*, and perceived stress*, but not cognitive reappraisal. | |  |
| **Psychological** | | | | | | | | | | | |
| After-  Deployment^b^ [85] | 13 weeks | | Quasi-  experimental design | | Student veterans with PTSD symptoms:  *N* = 11 | | 0% | Four participants showed a decrease in PTSD over the course of the intervention phase.* Two participants showed decreases in self-reported depression over the course of the intervention phase.* Three participants reported improved overall functioning over the course of the intervention phase.* | |  |  |
| Mobile Anger Reduction Intervention^a,b^ [89] | 4 weeks | | Mixed methods successive cohort design | | Veterans with PTSD and elevated anger; *N* = 13  (cohort 1 *n* = 5; cohort 2 *n* = 8) | | Cohort 1 = 0%  Cohort 2 = 23.5% | Participants reported that they were satisfied with the app and that it was helpful and easy to understand. The notifications received the lowest rating (notifications were not displayed for the first cohort). Mean pretreatment anger and hostile bias decreased at posttreatment, and mean pretreatment benign bias increased at posttreatment.  Cohort 1: All participants completed the 20 treatment sessions. Majority reported the app was easy to use, it helped them calm down, and it changed how they thought about situations. They reported the following weaknesses for the app: technical glitches, disruptive reminders, and over-repetition of scenarios (note: some enjoyed this). Participants enjoyed the frequency and length of sessions, the nightly diary, and some liked the My Progress sections which tracked progress and provided badges.  Cohort 2: 5/8 completed all 20 sessions (1 individual completed 5 sessions). Participants liked the simplicity and ease of use of the app. They like that they app “dinged” when they got an answer right, and that they could save progress and return to their question. Participants felt the app helped them change the way they thought about things and helped their angry thoughts. One participant interpreted the app as suggesting that it is wrong to feel anger, one felt nightly diaries were unhelpful, two did not understand the rationale of the treatment, two did not agree with the benign interpretations of the scenarios that were about inattention, and some noted app glitches. 3/6 reported they would have preferred to complete the treatment daily rather than 5 times a week. In response, the developers added 28 sessions, added more rationale for the treatment, added questions to the nightly diary, and added additional pop-up notifications. | |  |  |
| Building Resilience^b^ [62] | 3-9 months | | Repeated measures design: Randomly assigned to three cohorts (3-month; 6-month, or 9-month follow-up) | | PSP (paramedic students):  *N* = 34  (cohort 1: 3-month *n* = 8; cohort 2: 6-month *n* = 20; cohort 3: 9-month *n* = 6) | | 0% | Cohort 1 (3-month follow-up) showed a non-significant increase in resilience. Cohort 2 (6-month follow-up) showed a decrease in resilience scores*. Cohort 3 (9-month follow-up) showed a decrease in resilience.* For participants that completed all three phases of the study, the overall results suggest that mean resilience scores decreased from baseline to follow-up assessment.* | |  |  |
| COVID-19 Anxiety and Stress Resilience Training (COAST)^b^ [91] | 4 weeks | | Feasibility study | | PSP (mostly fire and ambulance workers):  *N* = 52 | | 0% | On average, users were satisfied with the program. Some reported difficulties using a web-based format or wanted more visualization of content. The self-efficacy module was used most often followed by mindfulness. First responders who had tested positive for COVID-19 and those who had been quarantined were more likely to engage in the program. There were confidentiality concerns among participants. | |  |  |
| Daily Coping Toolkit^b^ [87] | 1 week | | RCT: Low-dose versus high-dose intervention | | PSP (medical and emergency personnel, police, fire)  *N* = 28 | | 47% | Overall decrease in negative emotions* and increase in positive emotions. Post-hoc analysis revealed that the completion of positive mood prompts impacted overall improvement, those that did not complete these prompts (*n* = 13) showed no gains. Of those who completed two or more toolkit sessions (n=19), while controlling for a number of variables, experienced greater increase in positive emotions* and decrease in negative emotions. 70% of follow-up participants rated the intervention as having moderate to high effectiveness, and rated negative side effects as unlikely. | |  |  |
| Mental Health Toolkit for Veterans Project (MeT4VeT)^b^ [83] | Minimum of 28 days | | Feasibility and acceptability trial | | Veterans that served for the UK Armed Forces with mental health distress: *N* = 50 (intervention  *n* = 24; control  *n* = 26) | | 1 month:  25% intervention  15% control  3 months:  17% intervention 14% control | For the intervention group, symptoms of mental health distress (anxiety/dep)* and PTSD symptoms* decreased from pre-intervention to 3 month follow-up. For the intervention group, well-being* and psychological quality of life* improved from pre-intervention to 3-month follow-up. For the control group, there were no significant differences in any of the outcome measures. The intervention group showed improvement in psychological quality of life compared to control from baseline to 3-month follow-up*. No significant difference between groups for change in any other outcome measures baseline to 1 month and baseline to month 3. In terms of acceptability, the intervention group initialized the app a median of 8.5 times (control group = 2) over a period of 2.5 weeks (median; control group = 1 week). Median session duration was 20.7 seconds (control group = 29 seconds). The People section was accessed most frequently followed by the Dashboard section. Tracking was accessed by the least number of participants and used the most amount of times (79%). The Tools section was used for the least amount of time. The usability scores indicate positive responses to the app. | |  |  |
| Resilience@Work (RAW)^b^ [96] | 6 sessions | | Pilot Study: Quasi-experimental design | | Firefighters  *N* = 29 | | Sessions completed  1+: 100%  3+: 45%  5+: 52%  6: 62% | Non-significant positive changes in resilience, psychological flexibility, and experiential avoidance. | |  |  |
| RAW^b^ [67] | 6 sessions | | RCT: Randomization at the station level to RAW versus control | | Firefighters (12 stations):  *N* = 143 (RAW *n* = 60) | | Control:  6-weeks 54%  6-months 57%    Intervention:  6-weeks 32%  6-months 47% | Group-by-time interaction at 6-months revealed that intervention group increased in overall resilience after adjusting for baseline age, years of service, and number of traumatic incidents*. Non-significant difference between groups for bounce-back resilience, mindfulness, self-compassion, cognitive fusion, experiential avoidance, and sense of purpose in life at 6 weeks and 6 months. Compared to controls, the intervention group showed improvements in optimism*, use of instrumental support*, and use of emotional support* at 6 weeks, but not at 6 months. Compared to controls, the intervention group showed improvements in active coping* at 6 months, but not at 6-weeks. Compared to controls, RAW *completers* (those that completed 5-6 sessions) showed a positive change in overall resilience at 6-month follow-up*, and in mindfulness at 6-week* and 6-month* follow-ups. | |  |  |
| T2 Mood Tracker (T2)^a^ [72] | 2-3 weeks | | Proof-of-concept (quantitative and qualitative feedback) | | MM receiving care for physical or psychological injury:  *N* = 8 | | 0% | Participants rated T2 as “somewhat” to “very” easy to use. Seven participants found it very useful/beneficial, six participants said they would use T2 to track and share with their provider. A majority of participants reported that it was useful and created their own rating scales to track additional individual issues. One user found the multimedia capability T2 useful, “Used voice function for documenting because of my tremor” (p. 1455). | |  |  |

^a^DMHI costs money to access

^b^Not available in Canada, could not access, or no longer available
